# Model-Based Feedback Control for an Automated Micro Liquid Dispensing System Based on Contacting Droplet Generation through Image Sensing

**DOI:** 10.3390/mi14101938

**Published:** 2023-10-18

**Authors:** Qing Qian, Wenchang Xu, Haoran Tian, Wenbo Cheng, Lianqun Zhou, Jishuai Wang

**Affiliations:** 1 School of Biomedical Engineering (Suzhou), Division of Life Sciences and Medicine, University of Science and Technology of China, Hefei 230026, China; qianqing@sibet.ac.cn (Q.Q.); xuwc@sibet.ac.cn (W.X.); 2 CAS Key Laboratory of Biomedical Diagnostics, Suzhou Institute of Biomedical Engineering and Technology, Chinese Academy of Sciences (CAS), Suzhou 215163, China; tianhr@sibet.ac.cn (H.T.); chengwb@sibet.ac.cn (W.C.); 3 Tianjin Guoke Medical Engineering and Technology Development Co., Ltd., Tianjin 300399, China

**Keywords:** micro fluid dispensing system, image sensing, contacting droplet generation, feedback control

## Abstract

Over the past few decades, micro liquid dispensing technology has been widely used in biology, chemistry, material and environmental sciences due to its efficacy in processing multiple samples. For practical applications, precise and effective droplet generation is very important. Despite numerous droplet generation methods, the implementation of droplet-on-demand still faces challenges concerning system complexity, precision, cost, and robustness. In this work, a novel on-demand contacting droplet generation method incorporated with model-based feedback control with an image processing unit as a sensor was proposed. By studying droplet identification using image processing techniques, the model of droplet formation was simplified. Then model-based feedback control was implemented using volumes of dispensed samples as sensing signals by tuning related parameters adaptively to resist disturbances. The proposed method was integrated and applied to a homebuilt automated micro liquid dispensing system with droplets ranging from 20 nanoliter to 200 nanoliter. The experimental results demonstrated a high degree of accuracy and precision. Additionally, the proposed system’s practical utility was evaluated by analyzing mutations in genes associated with sensorineural hearing loss, verifying its effectiveness.

## 1. Introduction

In recent years, advances in liquid handling systems as a powerful tool for high-throughput assays have been witnessed in modern life science laboratories [1,2,3], especially nucleic acid sequencing, which often analyzes samples deposited onto target locations on substrates microarray [4,5]. Tests have been performed at the reactive points of microarray received genetic samples [6]. It is reported that about one-third of errors are generated during the sampling process by manual operations [7]. Therefore, there is a need for sampling instruments that can deposit samples in target locations [8]. Automatic liquid dispensing systems are necessary for the usage of dispensing samples to the target microarrayers precisely without contamination pollution.

**Figure 1:**
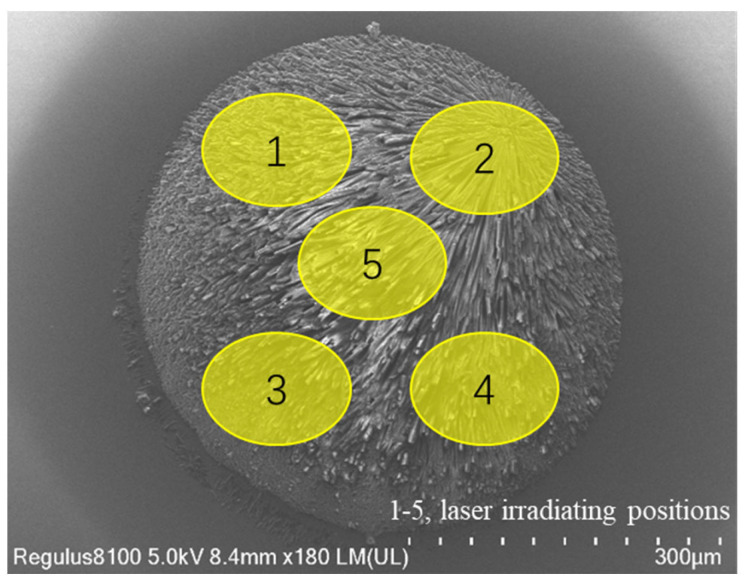
Close up of the chip with multiple irradiated positions.

There are many liquid handling systems developed recently [9,10,11]. Das et al. [12] presented a fluid dispensing system that had a range of 1 to 100 µL with an accuracy of 99.5% and a standard deviation of 150 nL. A peristaltic pump used independently should be calibrated because of the inaccurate and progressively lower volume due to the gradual deterioration of tubing elasticity. The system was based on a level sensing modular to control the volume of fluid before the peristaltic pump. The advantage of the proposed dispensing system was that the periodic calibration was not needed, because the peristaltic pump pulled the fluid to the intended application which eliminated the issue of lowering dispense volume over time. The individual components of the system can also be used for biomedical applications. Wu et al. [13] presented a high-throughput automated sample processing system based on the principle of air displacement with an 8-channel pipetting arm. The platform could process 96 samples with a capacity of 10 to 350 µL simultaneously while meeting the standards set by the ISO-8655 [14] and JJG 646-2006 [15]. Tong et al. [16] developed a nucleic acid analyzer with a microfluidic droplet-based real-time PCR assay integrated with an automated liquid handling robot. Nucleic acid analyzer is one kind of molecular diagnostic technique that involves nucleic acid extraction, amplification and detection. The proposed analyzer could achieve multiple operations including sample handling, nucleic acid extraction, PCR amplification and fluorescence detection of droplet array. The integrated system was adopted to a minimized size of 50 × 45 × 45 cm (length × width × height) with a cost reduction to CAD 900. In the past decades, Matrix assistant laser desorption ionization—Time of Flight Mass Spectrometer (MALDI-TOFMS) has been rapidly developed [17]. MALDI-TOFMS has been widely used for a variety of applications in biology, sequencing, environmental pollution monitoring, clinical diagnosis and other fields [18,19,20,21]. Before the analytical process, liquid handling is necessary to transfer nanoliter samples to chip arrays with substrates on a dots matrix. After samples and substrates co-crystallize, analytes are irradiated by the laser of a MALDI-TOFMS that induces desorption and ionization of the molecules. Data are collected from multiple positions of a crystal for an average intensity value as shown in Figure 1.

If the dispensing volume is too large, the signal of the detector will be oversaturated, which will affect the sensitivity of the detection; otherwise, it may result in poor responsiveness, even undetectable. Hence the volume of the dispensing liquid should be appropriate to cover the whole substrate dot to ensure co-crystallization without redundancy. Although an integrated robotic sample transfer device that could process fluid samples for mass spectrometry was invented, the system was open-loop controlled, leading to droplets not being generated precisely enough [22]. A visual image recognition system was widely used to control the on-demand movements of motors to reduce positioning errors [23,24,25,26]. Hence, an automated droplet-on-demand dispensing system with closed-loop feedback control is required for the sample preparation of the MALDI-TOFMS [27].

In this paper, a home-built, automated micro liquid dispensing system was developed and validated for the preparation of samples. The system can deliver precise amounts of small volumes, particularly nanoliter volumes, based on contacting droplet generation. An adapted controller was designed with the image process unit as the sensing signal. The control model ensured the volume to be dispensed precisely despite the liquid properties. The results showed that the proposed system was able to carry out sample preparations with reliability and robustness for the practical use of MALDI-TOFMS.

## 2. Materials and Methods

**Figure 2:**
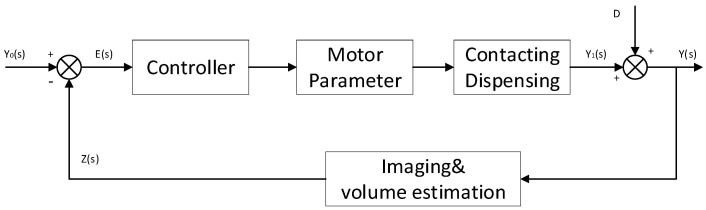
The block diagram of the feedback control system.

The proposed system contains three main units: the imaging-based unit, the signal processing unit and the feedback control unit [28]. Figure 2 shows the block diagram of the control system. The contacting dispensing object has a pin tool on the *z*-axis motor end with a camera adjacent to it. In this work, the camera is used for capturing the droplet shape of the dispensed liquid. A computer is embedded as the signal processing unit, which calculates the volume of the dispensed droplet based on the area obtained by the imaging unit. The image processing unit is used as the sensor of the control system. The feedback control unit was executed by the embedded computer regulating the downward velocity of the *z*-axis motor through the Controller Area Network (CAN). The control strategy aims to minimize the error between the set-point volume of the droplet and the measurement obtained by the sensor, which is the image processing unit.

### 2.1. Momentum-Driven Droplet Image Acquisition

**Figure 3:**
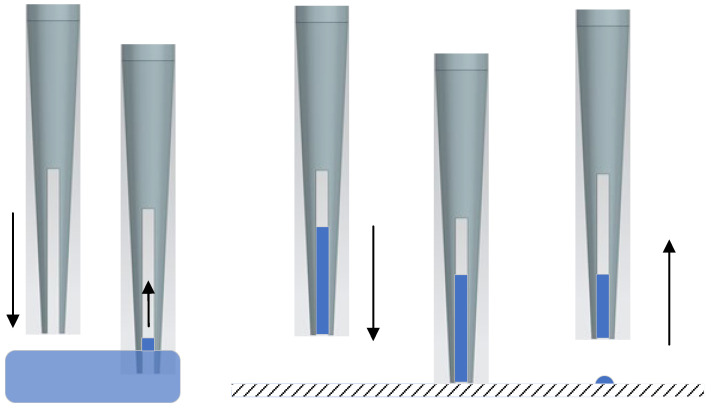
Dispensing process of the pin.

A solid coated pin has a slotted end, as shown in Figure 2. First, the pin is immersed into samples. Then, the pin is moved upwards. During this process, the pin could hold a liquid droplet with surface tension. After picking up samples during dipping, the coated pin is then transferred to the top of a target surface. If the pin is properly positioned, it will move downward to the target surface at a predetermined speed to impart a downward momentum to the liquid drawn by the pin. The pin is halted in the travel when contacting the aimed surface. The liquid samples keep moving at their imparted speed because of the downward momentum, therefore, samples are expelled from the slot to the target surface. After lifting the pin, the liquid samples are deposited at the target location. The process is shown in Figure 3. The dipping depth of the pin could be selected to obtain different quantities of samples. The downward velocity is varied according to the dispensing volume.

**Figure 4:**
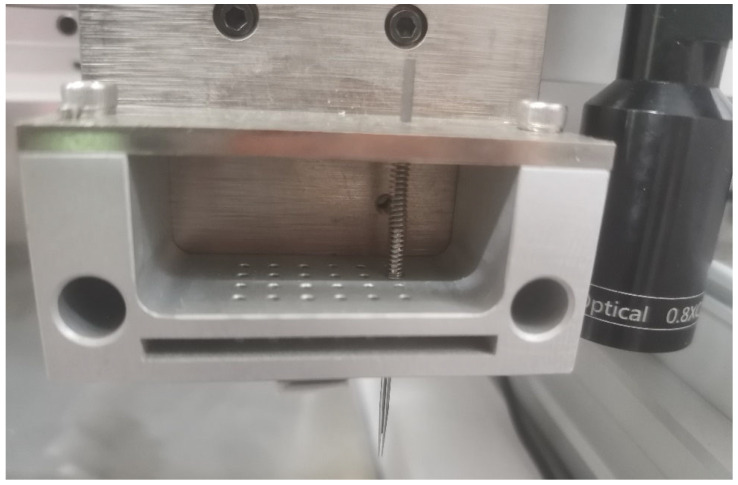
The overall structure of the pin tool.

Pins are installed on the pin tool which is embodied in a three-axis robotic-positioning assembly. Springs fixed to the pin are used for cushioning the upward movement when being compressed against the upper wall. The volume of the liquid sample is related to the descent speed and dipping depth of the pin in the sample with the nature of its volatility, viscosity and the like. The overall structure of the pin tool is shown in Figure 4. A camera set beside the pin tool captures the image of the dispensed droplet as soon as the pin moves to the initial *y*-axis position.

### 2.2. Image Pre-Processing

The median filter is one of the predominant filters for smoothing signals [29]. It has the advantage of simplicity and the ability to maintain edges which have led to extensive applications in the domain of image processing [30].

(1) $$g(x,y)=med\left\{f(x-k,y-l),(k,l\in W)\right\}$$

where $f\left(x,y\right)\;$ identifies the original image and $g\left(x,y\right)\;$ represents the estimated image. *W* is the window slide, which is processed as it slides through the image from the left edge to the right. In this work, a 10 × 10 window was used.

An algorithm based on the principle of least squares was used to obtain a suitable threshold (OTSU) [31]. The basic idea of OTSU is to divide the histogram into two groups with a certain gray level as the threshold, and calculate the variance between the two groups. When the variance is maximized, the image is divided into two groups with the corresponding threshold.

Let the pixels of the given image be represented in L gray levels [1, 2, …, *S*]. The total number of pixels is N:

(2) $$N=\sum_{i=1}^{S}n_{i}$$

The gray-level histogram is regarded as a probability distribution:

(3) $$p_{i}=n_{i}/N,\;p_{i}\geq 0,\sum_{i=1}^{S}p_{i}=1$$

the pixels then are divided into two classes *C*_0_ and *C*_1_ by a threshold at level *k*. The probabilities of class occurrence are given as follows:

(4) $$\omega_{0}=\mathrm{Pr}(C_{0})=\sum_{i=1}^{k}p_{i}=\omega \left(k\right)$$

(5) $$\omega_{1}=\mathrm{Pr}(C_{1})=\sum_{i=k+1}^{S}p_{i}=1-\omega \left(k\right)$$

where *C*_0_ denotes pixels with level [1, …, *k*] and *C*_1_ denotes pixels with level [*k* + 1, …, *S*]. Means of the two classes are given as follows:

(6) $$\mu_{0}=\sum_{i=1}^{k}i\;\mathrm{Pr}(i|C_{0})=\sum_{i=1}^{k}ip_{i}/\omega_{0}=\mu (k)/\omega \left(k\right)$$

(7) $$\mu_{1}=\sum_{i=k+1}^{S}i\;\mathrm{Pr}(i|C_{1})=\sum_{i=k+1}^{S}ip_{i}/\omega_{1}=\frac{\sum_{i=1}^{S}ip_{i}-\mu (k)}{1-\omega (k)}$$

the mean gray level of the histogram at threshold *k* is given:

(8) $$\mu (k)=\sum_{i=1}^{k}ip_{i}$$

for any choice of *k*, the following relation can be verified as:

(9) $$\omega_{0}\mu_{0}+\omega_{1}\mu_{1}=\mu_{1}(S)=\sum_{i=1}^{S}ip_{i}\;\;\;\;\;\;\;\;\omega_{0}+\omega_{1}=1$$

The discriminant criterion is used to evaluate the threshold at level *k*, where:

(10) $$\delta_{B}^{2}=\omega_{0}\omega_{1}{\left(\mu_{1}-\mu_{0}\right)}^{2}$$

The optimal threshold k* maximizes $\delta_{B}^{2}$, so the maximum is sought as follows:

(11) $$M^{*}=\left\{k;\omega_{0}\omega_{1}=\omega_{k}[1-\omega_{k}]>0,\;\mathrm{or}\;0<\omega_{k}<1\right\}\;\;\;\;\;\;\;\;k\in M^{*}$$

In a binary image, the object consists of non-zero pixels and 0 pixels as the background. B is a P × 1 cell array, P is the number of objects, and each cell is Q × Matrix of 2, corresponding to the coordinates of the object outline pixels. Each row in Q represents the location coordinates of the boundary pixels of the connected body (the first column is ordinate Y, the second column is transverse X), and Q is the number of boundary pixels. The outline of a binary graph object, including the outer outline and the inner edge, is obtained using the above algorithm. After the outline is recognized, the dispensing areas are calculated with pixels as units.

**Figure 5:**
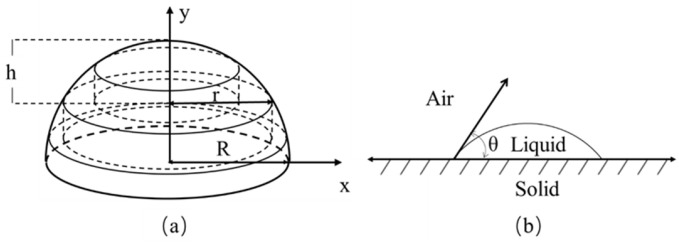
(**a**) Spherical cap in multi-layer segmentation. (**b**) Schematic diagram of the contact angle.

The shape of the dispensed fluid could be seen as a spherical cap composed of multi-layer stacked cylinders with radius *r* and height *y*, as shown in Figure 5.

The function of the estimated volume using an approximation in multi-layer segmentation is as follows:

(12) $$V=\int_{R-h}^{R}\pi r^{2}dy=\pi R^{2}h-\frac{1}{3}\pi h^{3}$$

where *V* is the volume of the dispensed fluid, *R* is the radius of the sphere and *h* is the height of the spherical cap. If the contact angle of the dispensed fluid is *θ*, then:

(13) $$R=\frac{R_{0}}{\sin\theta }$$

(14) $$h=R\left(1-\cos\theta \right)$$

the volume of the dispensed fluid is:

(15) $$V=\frac{\pi }{3}{\left(\frac{D_{ref}}{D_{pix}}R_{0}\right)}^{3}\left(\cos^{3}\theta -3\cos\theta +2\right)$$

where *D_ref_* is the distance in mm between two adjacent referent points, *D_pix_* is the total number of pixels between two adjacent referent points and *R*_0_ is the radius in pixels of the dispensed area. Specially, when the contact angle is 90°, the volume is equal to that of a hemisphere. The whole process has been implemented successively during the dispensing procedure.

### 2.3. Incorporation with Feedback Control

The liquid dispensing system process is as follows: the pin dips into a reservoir of samples, draws liquid up into the pin tool, then travels to a certain position, and moves downward to the microchip at a predefined speed. The sample is expelled onto the surface of the microchip by the momentum of the liquid resulting from the abrupt halting. The next round of transfers is executed after the cleaning step is finished.

It has been found that the volume of the liquid sample expelled has a direct proportion to the downward velocity and dipping depth of the slotted pin in samples. The function is as follows:

(16) Volume=f(v,h) v∈V,h∈H

where *v* is the velocity and *h* is the depth.

## 3. Results

**Figure 6:**
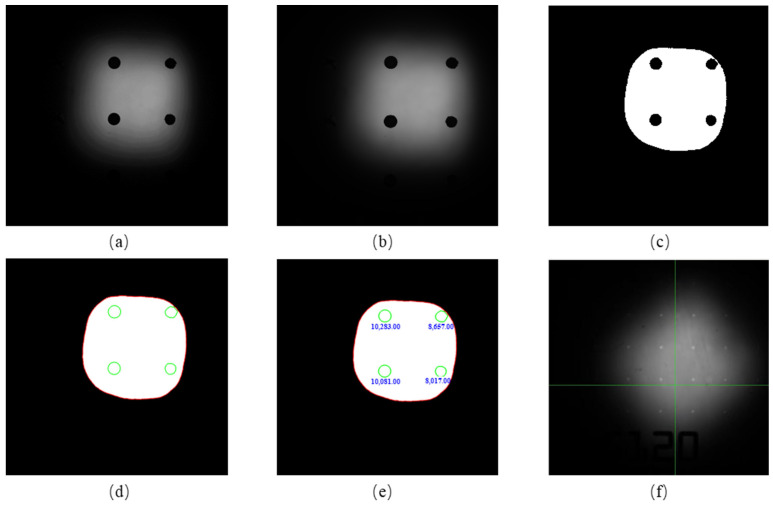
Image pre-processing: (**a**) Original image of the dispensing droplets. (**b**) Image after filtered. (**c**) Image after OSTU. (**d**) The outline of the droplet. (**e**) Areas of the liquid droplets (pixel). (**f**) Image of reference points.

The image pre-processing results are shown in Figure 6. The original image is Figure 6a. The filtered image is shown in Figure 6b. It can be seen that the grayscale gradient of Figure 6a is missing. Figure 6c shows the results of the application to the filtered image. The outer outline and the inner edge are obtained using the above algorithm and shown in Figure 6d. After the outline is recognized, the areas are calculated with pixels as units, as shown in Figure 6e. The whole process has been implemented successively during the dispensing procedure. To determine the volume of the dispensed fluid, an image of reference points was taken to obtain *D_ref_* and *D_pix_*.

**Figure 7:**
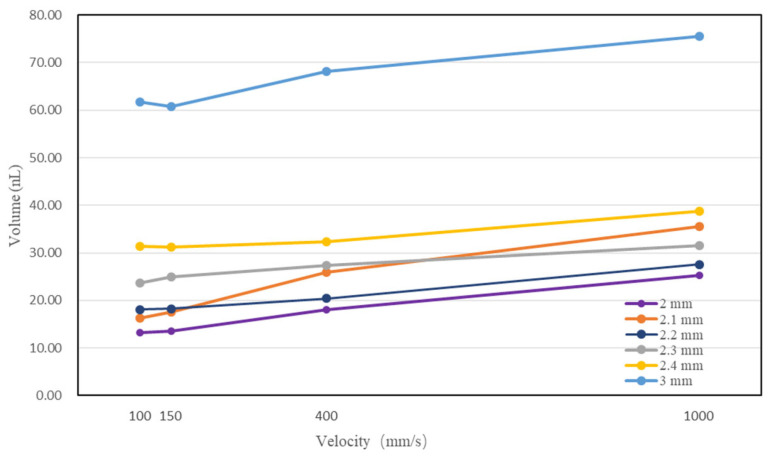
The relationship between velocity, dipping depth and dispensing volume.

To find the influencing factors of the volume, several experiments were carried out. A high-speed camera (OLYMPUS i-SPEED CDU) was used to capture the side image of a droplet to obtain the contact angle. Six different dipping depths of the sample were applied with various velocities. In this case, pure water was used as the sample, *D_ref_* was 1.2 mm and *D_pix_* was 260 pixels. As the contact angle was about 90°, the volume was calculated as a hemisphere. The detailed parameters, averages of results and their Standard Deviation (SD) are listed as follows in Table 1:

The average and standard deviation (SD) values of experiments using the same speed of 100 mm/s for a given dipping depth are listed in Table 2:

The relationships between the proposed downward velocity and volumes of different dipping depths are shown in Figure 7. As the velocity increased, the dispensed volume increased. At the same velocity, most dispensing volumes became larger as the pin was immersed deeper.

Samples of the automated micro liquid dispensing system are DNA/RNA extension products made by PCR (Polymerase Chain Reaction). There are mainly two types of fluid used by PCR, including oil phase buffer and droplet discrete phase. The commonly used oil phase buffer includes dimethyl silicone oil, fluorinated oil, mineral oil, glycerol, etc. Discrete phase is pure water or buffer commonly. To discuss the influence of the density and viscosity in dispensing process, pure water, calibrator, two kinds of samples were dispensed. Properties of them at room temperature 25 °C are listed in Table 3.

Four kinds of fluid were dispensed at a velocity of 1000 mm/s with a dipping depth of 3 mm for ten times. Dispensing areas are listed in Table 4 in pixels.

**Figure 8:**
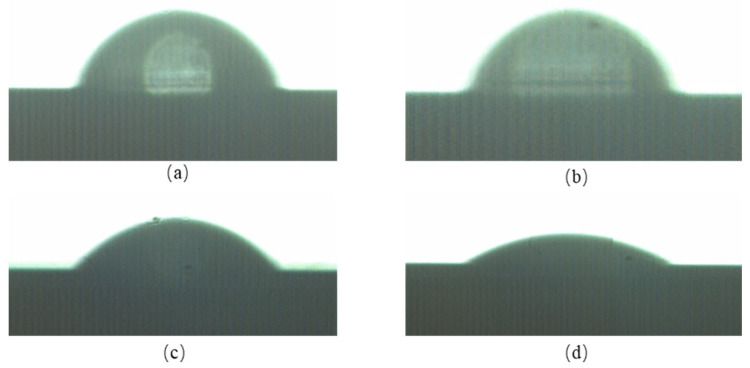
Side views of droplets: (**a**) Pure Water. (**b**) Calibrator. (**c**) Sample 1. (**d**) Sample 2.

Dispensing areas of four kinds of liquid were similar, but differences were observed in side views of droplets as shown in Figure 8.

The contact angles are 86.3°, 86.5°, 34.2° and 15.1°. After data fitting, contact angles of fluids with different densities and viscosities were obtained. As the density and viscosity increased, the contact angle of the droplet decreased, which led to a decrease in dispensing volumes, as shown in Table 5. As each droplet covered the whole substrate area, there was no impact on final mass spectrometry detection.

**Figure 9:**
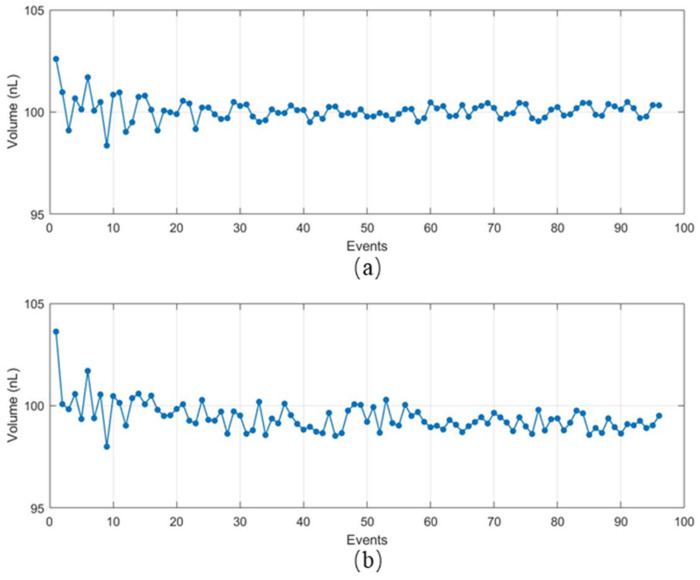
(**a**) Response of feedback control. (**b**) Response of open-loop control.

Experiments were conducted to study the response time of the model-based feedback control system and open loop system in Matlab 2021b using pure water. The experimental results can be referred to as Figure 9. This study indicates that the droplet generation assisted by model-based feedback control has a shorter response time and lower deviation compared to conventional passive droplet generation methods assisted by open loop control. In addition, we have tested more different setpoint volumes and compared the response performance of the model-based feedback control system and the open loop control system. The feedback control system can adjust the dipping depth adaptively so that the volume is about 100 nL. Without feedback control, the dipping depth is constant while the level of samples becomes lower after several tests, so the volume of the dispended fluid is less than 100 nL gradually. The robustness of our developed system is better.

To verify the proposed algorithm, a homebuilt automated liquid dispensing system was developed and discussed in the next section.

## 4. Discussion

**Figure 10:**
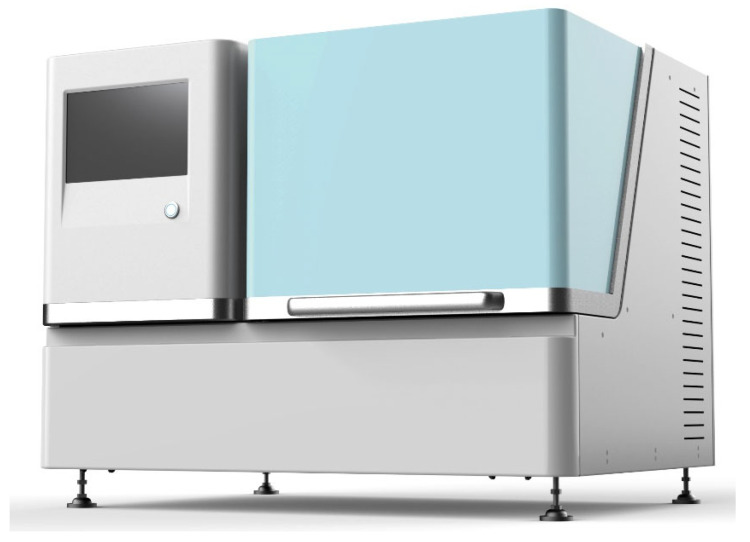
Schematic diagram of the home-built system.

The overall appearance of the system is shown in Figure 10. The pin is designed for dispensing liquid with the range of 20 nL to 200 nL.

**Figure 11:**
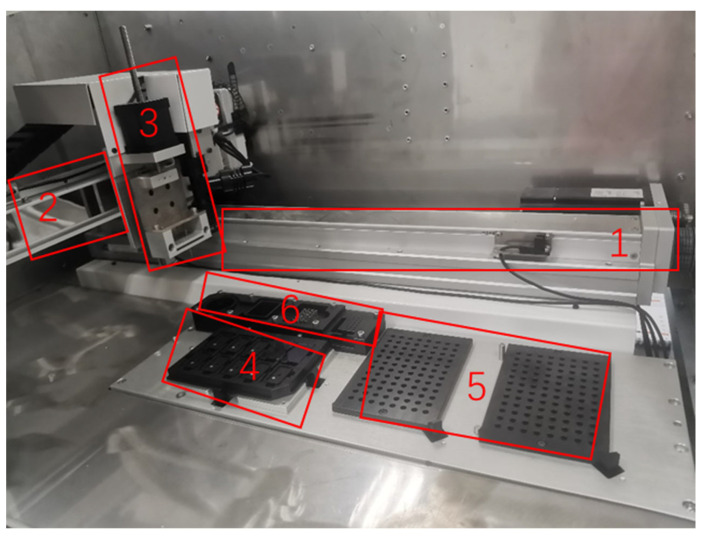
Details of home-built system 1. *X*-axis robot 2. *Y*-axis robot 3. *Z*-axis robot with the pin tool in the end 4. Chip location 5. Sample tray 6. Wash station with tanks under the platform.

Users operate the system through the touch screen on the left side. The power switch and other interfaces are on the back of the instrument. The door on the right side of the instrument can be opened. The layout inside the door is shown in Figure 11.

**Figure 12:**
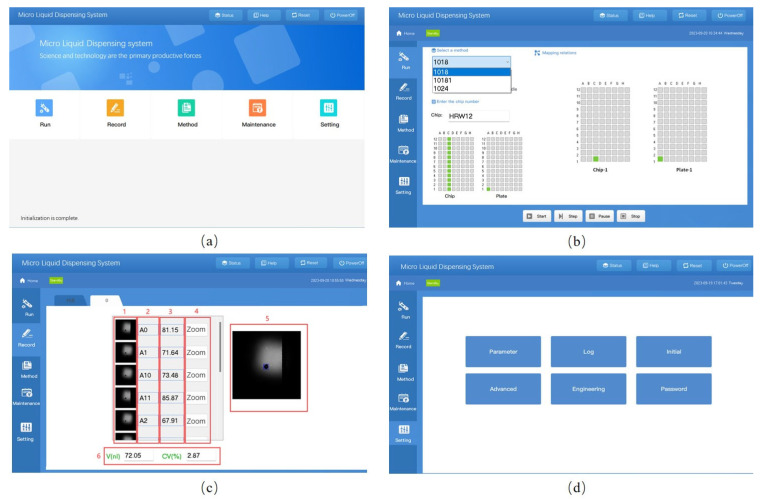
Software interface: (**a**) Initial page. (**b**) Method page. (**c**) Setting page. (**d**) 1. Image list; 2. Index number on a chip; 3. Dispensed volume (nL); 4. Zoom; 5. Image details; 6. Statistical information.

The system consists of a three-axis robotic arm with the pin tool on the *z*-axis, a wash station, a sample supply site and the dispensed chip area. As in Figure 11, the *X*-axis and *Y*-axis robot achieve the movements of the robotic arm in the horizontal direction with a repeatability of 10 µm, so it can precisely move pins onto the points of the microchip and sites of the samples. The *Z*-axis robotic arm leads the pin tool moving up and down with a repeatability of 1 µm. The accelerated velocity, retarded velocity and velocity of the three-axis robotic arms are all adjustable. Parameters should be tuned appropriately before micro fluid dispensing. The camera set on the *Z*-axis robotic arm is fixed, which has the function of autofocus. It captures images of dispended fluid automatically with the target site number stored while dispensing. The fourth part of the system is the chip location which accommodates up to 10 chips at the same time. The sizes of locations are consistent. The fifth part of the system is the sample tray which is capable of accommodating double 96-well plates. The wash station includes a diaphragm pump, clean and waste tanks, and a drying site with a vacuum pump. All parts of elements are controlled by a computer through a user interface software as shown in Figure 12.

The initial page of the software is shown in Figure 12a. The main functions in the initial page are Run, Record, Method, Maintenance and Setting accordingly. After the selection of the main function, the software will jump to the page of the corresponding function with main functions listed on the left slide. The function of Run is to display the dynamic process of dispensing. The function of Record is shown in Figure 12d. Column 1 is the list of images of dispensing fluid, column 2 is the index of the target chip number, column 3 is the list of volumes, and column 4 is the function of zoom, which shows the enlarged image in column 5 if users want to seek more details. Part 6 of the record page is statistical information about the microfluid, including average volume and covariance. The Method page is shown in Figure 12b. In this page, corresponding relationships are created with a given method name. Methods could be found and revised as well as deleted using buttons at the bottom of the page. The function of Maintenance is used to pour water into tanks or drain waste with operation instructions. The Setting page in Figure 12c has the following functions: changing parameters, log view, initialization, advanced parameters setting, user management and password management. Passwords are only required when operating advanced parameters. Shortcuts on the top right of the initial page are State, Reset and Shut down. The state button will be flashing if the device is abnormal. After pressing the reset button, all parts of the device will undergo initialization. Shut down will be confirmed before all parts of the system be switched off.

The whole working process is as follows: The user first predetermines a method in the software with a set of transfer parameters. For example, the positions of samples located and the aimed chip map to be dispensed, as well as the volume of the liquid spot. The image data of fiducial marks are processed by the processing unit to determine the precise position of each sample deposition site on the chip. Then, a standard cleaning cycle is initiated which includes rinsing in the wash station and drying in the vacuum drying station. After that, the transfer process could be displayed. Samples are transferred from the sample well to chips while a live video as well as a graphic of the transfer status of the sample deposition are shown. The image of the dispensing sample and the information of the image are displayed. The model-based feedback control unit ensures that the volume is closed to the set value automatically, as the levels of the sample and distances between the pin tool and chip are changeable. There are redundant target spots on the chip to overcome the response time of the control strategy.

The volume of the dispended fluid has a range of 20 nL to 200 nL. To determine the proper volume to be used, the calibrator was tested using an Agena MassARRAY CPM96 (Agena Bioscience, San Diego, CA, USA). Different on-demand volumes were dispensed five times by 20 nL as a step from 20 nL to 200 nL. Results are shown in Table 6.

**Figure 13:**
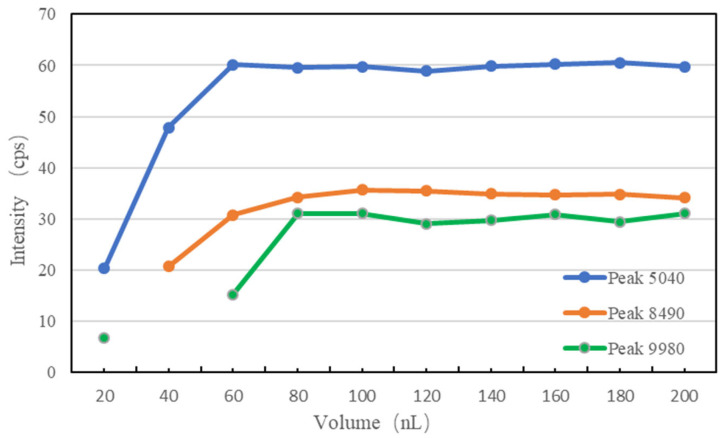
MS profiles of the calibrator with peak information.

The corresponding curves are shown in Figure 13. When the volume was 20 nL, the peak of mass 8490 failed to appear. When the volume was 40 nL, the peak of mass 9980 was lost. As the dispensing volume became larger, intensities grew accordingly. When the volume was less than 60 nL, the intensity was lower than the target value of the calibrator, indicating that the co-crystallization was not enough. When the volume was greater than 80 nL, all intensities were relatively constant. Because the substrate was constant, the dispensing volume had to be larger than 80 nL to ensure that the samples and substrates were adequately mixed. In this study, 100 nL was selected as the default dispensing parameter.

When the dispensed volume was set as 100 nL, results and the corresponding Cross-Validations (CVs) of the dispensed chip are shown in Table 7.

**Figure 14:**
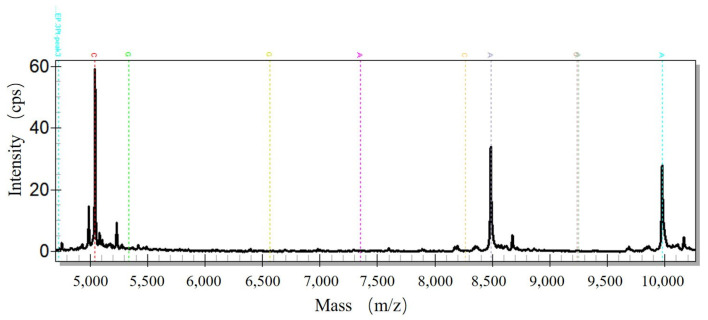
Mass spectra of the calibrator.

The dispensed samples all presented active peaks, and their CVs were less than 5%, which met the related standard. One of the mass spectrograms profile is shown in Figure 14 which illustrated the distribution of peaks according to the mass-to-charge ratio.

Hearing loss (HL) is the most common sensory disorder, affecting one child in every 500 to 1000 at birth [32]. Genetic causes are estimated to represent 50–70% of congenital deafness cases. Most hereditary HL cases are caused by mutations in nuclear genes [33]. In this case, the deafness gene GJB2 is taken as the detection object. A total of 15 samples of PCR products were prepared, of which there were 2 samples with a known mutation site at GJB2_235delC. Samples were processed by the homebuilt system and results were investigated by Agena MassARRAY (Agena Bioscience, San Diego, CA, USA) for the simultaneous analysis of mitochondrial alterations.

It can be seen from Table 8 that the home-built dispensing system with image processing feedback control algorithm proposed in this paper can not only better perform CV, but can also accurately identify the location of mass peaks of the mutation sample, which has a very important clinical application value.

## 5. Conclusions

In this paper, the development and validation of a home-built automatic micro liquid dispensing system was introduced. A model-based feedback control algorithm was proposed for droplet dispensing. First, a real-time imaging process was investigated to obtain the volume of the droplet. Then, the relationships between downward velocity and dipping depth and volume were found. With this knowledge, a model-based control system was built with the imaging unit as the sensor. Several experiments were carried out on the home-built micro liquid handling system. Compared with open-loop control, the proposed algorithm achieved higher system response and lower steady-state error, which can greatly improve the accuracy of dispensing volume. Based on the research of this paper, advanced control strategies will be considered to improve the robustness of this algorithm.

## Figures and Tables

**Table 1:** Results with different dispensing parameters.

Velocity (mm/s)	Volume (nL)					
	Dipping Depth 2 mm	Dipping Depth 2.1 mm	Dipping Depth 2.2 mm	Dipping Depth 2.3 mm	Dipping Depth 2.4 mm	Dipping Depth 3 mm
100	13.27	16.26	18.10	23.75	31.42	61.72
150	13.53	17.52	18.25	25.00	31.26	60.79
400	18.09	25.89	20.45	27.31	32.27	68.18
1000	25.25	35.57	27.57	31.57	38.75	75.53

**Table 2:** Average and Standard Deviation (SD) for different dipping depths.

	Volume (nL)					
	Dipping Depth 2 mm	Dipping Depth 2.1 mm	Dipping Depth 2.2 mm	Dipping Depth 2.3 mm	Dipping Depth 2.4 mm	Dipping Depth 3 mm
1	13.15	16.42	18.11	23.64	31.45	61.79
2	12.89	16.27	17.94	23.75	31.51	61.91
3	13.12	16.33	18.24	23.91	31.27	61.58
4	13.21	16.24	17.91	23.77	31.36	61.66
5	13.23	15.99	18.06	23.52	31.23	61.73
6	12.94	16.04	18.25	23.73	31.35	61.89
7	13.29	16.15	18.04	23.89	31.57	61.70
8	12.77	16.29	18.27	23.66	31.47	61.69
9	13.36	16.18	18.16	23.93	31.39	61.52
10	13.45	16.36	18.33	23.55	31.55	61.61
Average	13.14	16.23	18.13	23.74	31.42	61.71
SD	0.22	0.14	0.14	0.15	0.11	0.13

**Table 3:** Properties of four kinds of fluid.

Fluid	Density (Kg/m^3^)	Viscosity (Pa·s)
Water	997.04	0.0009
Calibrator	996.81	0.0009
Sample 1	1134.91	0.0013
Sample 2	1467.62	0.0024

**Table 4:** Results of different kinds of fluid.

	Volume (nL)			
	Pure Water (Pixels)	Calibrator (Pixels)	Sample 1 (Pixels)	Sample 2 (Pixels)
1	15,831	16,403	16,144	16,308
2	15,869	16,164	15,757	16,205
3	15,912	15,922	16,417	15,838
4	16,006	16,193	15,792	16,035
5	15,918	16,404	16,080	16,173
6	16,298	16,016	16,292	16,227
7	16,431	16,167	16,242	15,923
8	15,869	16,084	15,883	16,367
9	15,933	15,979	16,344	15,827
10	15,957	16,127	15,899	16,061
SD	199.5083958	161.5057275	239.7086194	190.6580418

**Table 5:** Volumes of different kinds of fluid.

	Volume (nL)			
	Pure Water (Pixels)	Calibrator (Pixels)	Sample 1 (Pixels)	Sample 2 (Pixels)
1	71.32	70.07	35.25	20.03
2	70.27	69.77	35.40	20.10
3	70.18	69.75	35.31	19.98
4	70.23	69.83	35.40	20.24
5	70.12	69.91	35.38	20.08
6	70.24	70.02	35.29	20.16
7	70.39	69.71	35.52	20.09
8	70.08	69.91	35.14	20.12
9	70.37	69.76	35.21	19.96
10	70.07	69.87	35.45	20.27

**Table 6:** Results of calibrator tests for different volumes.

Volume (nL)	Intensity (cps)		
	Mass 5040 (*m*/*z*)	Mass 8490 (*m*/*z*)	Mass 9980 (*m*/*z*)
20	20.32	NULL	6.77
40	47.89	20.68	NULL
60	60.15	30.76	15.24
80	59.56	34.26	31.06
100	59.71	35.64	31.10
120	58.92	35.46	29.08
140	59.85	34.87	29.69
160	60.18	34.73	30.87
180	60.52	34.84	29.38
200	59.73	34.12	31.04

**Table 7:** MS chromatographic results.

Sample No.	Intensity (*m*/*z*)			Volume (nL)
	Mass 5040 (*m*/*z*)	Mass 8490 (*m*/*z*)	Mass 9980 (*m*/*z*)	
1	58.88	34.57	29.45	101.97
2	57.63	35.52	30.21	101.57
3	60.15	35.02	30.17	99.78
4	59.56	34.32	29.91	101.73
5	59.71	35.29	30.89	102.87
6	58.92	35.97	30.98	100.04
7	59.85	35.20	29.63	103.71
8	60.18	34.45	29.72	100.65
9	60.52	34.42	30.82	101.52
10	59.73	35.16	29.31	103.01
CV (%)	1.41	1.14	2.24	1.26

**Table 8:** HL genetic chromatographic results.

Sample No.	Intensity (cps)				Volume (nL)
	Mass 6875 (*m*/*z*)	Mass 6960 (*m*/*z*)	Mass 7105 (*m*/*z*)	Mass 7068 (*m*/*z*)	
1	43.21	49.23	23.38	NULL	101.42
2	49.42	42.48	29.41	NULL	102.55
3	43.41	40.66	29.67	NULL	99.72
4	40.15	42.11	30.92	NULL	100.52
5	45.67	46.77	29.48	28.68	100.23
6	45.55	42.06	31.03	NULL	100.04
7	44.35	46.43	31.10	NULL	100.6
8	47.87	49.52	29.13	NULL	102.38
9	49.53	49.78	30.58	NULL	102.86
10	40.32	48.07	30.09	NULL	100.99
11	47.08	44.23	29.45	NULL	100.12
12	41.23	43.77	29.19	NULL	100.51
13	48.98	42.91	30.74	25.33	101.68
14	48.76	47.74	29.90	NULL	99.72
15	49.48	41.35	30.30	NULL	101.81
CV (%)	1.41	1.14	2.24		1.26

## Data Availability

The data that support the findings of this study are available from the corresponding author, upon reasonable request.

## References

[1] Bijarchi M.A., Favakeh A., Alborzi S., Shafii M.B. (2020). Experimental investigation of on-demand ferrofluid droplet generation in microfluidics using a Pulse-Width Modulation magnetic field with proposed correlation. Sens. Actuators B Chem..

[2] Zeng W., Yang S., Liu Y., Yang T., Tong Z., Shan X., Fu H. (2022). Precise monodisperse droplet generation by pressure-driven microfluidic flows. Chem. Eng. Sci..

[3] Fadaei M., Majidi S., Mojaddam M. (2023). Droplet generation in a co-flowing microchannel influenced by magnetic fields applied in parallel and perpendicular to flow directions. J. Magn. Magn. Mater..

[4] Thaysen J., Marie R., Boisen A. Cantilever-based bio-chemical sensor integrated in a microliquid handling system. Proceedings of the 14th IEEE International Conference on Micro Electro Mechanical Systems.

[5] Coelho B.J., Neto J.P., Sieira B., Moura A.T., Fortunato E., Martins R., Baptista P.V., Igreja R., Águas H. (2023). Hybrid Digital-Droplet Microfluidic Chip for Applications in Droplet Digital Nucleic Acid Amplification: Design, Fabrication and Characterization. Sensors.

[6] Mcdowall R.D. (2005). SAMPLE HANDLING. Automated Sample Preparation. Encycl. Anal. Sci..

[7] Noparatayaporn P., Sakulbumrungsil R., Thaweethamcharoen T., Sangseenil W. (2017). Comparison on Human Resource Requirement between Manual and Automated Dispensing Systems. Value Health Reg. Issues.

[8] Takken M., Wille R. (2022). Simulation of Pressure-Driven and Channel-Based Microfluidics on Different Abstract Levels: A Case Study. Sensors.

[9] Torres-Acosta M.A., Lye G.J., Dikicioglu D. (2022). Automated liquid-handling operations for robust, resilient, and efficient bio-based laboratory practices. Biochem. Eng. J..

[10] Lee J., Koen V., Gooris G., O’Mahony C., Jiskoot W., Bouwstra J. (2021). Engineering of an automated nano-droplet dispensing system for fabrication of antigen-loaded dissolving microneedle arrays. Int. J. Pharm..

[11] Jia M., Wu M., Li Y., Xiong B., Wang L., Ling X., Cheng W., Dong W.-F. (2022). Quantitative Method for Liquid Chromatography–Mass Spectrometry Based on Multi-Sliding Window and Noise Estimation. Processes.

[12] Das C., Wang G., Nguyen C. (2017). A Low-Cost, Accurate, and High-Precision Fluid Dispensing System for Microscale Application. J. Lab Autom..

[13] Wu Y., Chen H., Wang W., He N., Liu B. (2016). Development and Validation of an Automated Liquid Handling System for Sample Preparation Based on Multichannel Air Displacement Pipetting Technology. J. Nanosci. Nanotechnol..

[14] (2022). Piston-Operated Volumetric Apparatus.

[15] (2006). Verification Regulation of Locomotive Pipette.

[16] Lin T.T., Wang J.W., Shi Q.N., Wang H.F., Pan J.Z., Fang Q. (2023). An automated, fully-integrated nucleic acid analyzer based on microfluidic liquid handling robot technique. Anal. Chim. Acta.

[17] Wu A., French D. (2013). Implementation of liquid chromatography/mass spectrometry into the clinical laboratory. Clin. Chim. Acta.

[18] Luo Y., Zheng Z., Zheng X., Li Y., Che Z., Fang J., Xi L., Nguyen N.T., Song C. (2022). Model-based feedback control for on-demand droplet dispensing system with precise real-time phase imaging. Sens. Actuators B Chem..

[19] Wiley W.C., Mclaren I.H. (1955). Time-of-Flight Mass Spectrometer with Improved Resolution. Rev. Sci. Instrum..

[20] Strupat K., Karas M., Hillenkamp F. (1991). 2,5-Dihydroxybenzoic acid: A new matrix for laser desorption—Ionization mass spectrometry. Int. J. Mass Spectrom. Ion Process..

[21] Nielen M., Hooijerink H., Zomer P., Mol J. (2011). Desorption electrospray ionization mass spectrometry in the analysis of chemical food contaminants in food. Trends Anal. Chem..

[22] Silbert R., Capella R., Cuzens J. (2015). Integrated Robotic Sample Transfer Device. U.S. Patent.

[23] Niemitz L., van der Stel S.D., Sorensen S., Messina W., Venkata Sekar S.K., Sterenborg H.J.C.M., Andersson-Engels S., Ruers T.J.M., Burke R. (2023). Microcamera Visualisation System to Overcome Specular Reflections for Tissue Imaging. Micromachines.

[24] Khlynov R.D., Ryzhova V.A., Yarishev S.N., Konyakhin I.A., Korotaev V.V., Shelepin Y.E., Djamiykov T.S., Marinov M.B. (2022). Analysis of Polarization Images in the Microphysical Blood Parameters Research for the Hematocrit Diagnostics. Micromachines.

[25] Wang Y., Jia H., Jia P., Chen K. (2023). An Automatic Detection Method for Cutting Path of Chips in Wafer. Micromachines.

[26] Li K., Kinoshita Y., Sakai D., Kawano Y. (2023). Recent Progress in Development of Carbon-Nanotube-Based Photo-Thermoelectric Sensors and Their Applications in Ubiquitous Non-Destructive Inspections. Micromachines.

[27] Chien C.-Y., Yang Y., Gong Y., Yue Y., Chen H. (2022). Blood-brain barrier opening by individualized closed-loop feedback control of focused ultrasound. BME Front..

[28] Soto M.G., Adeli H. (2017). Recent advances in control algorithms for smart structures and machines. Expert Syst..

[29] Tukey J.W. (1977). Exploratory Data Analysis.

[30] Appiah O., Asante M., Hayfron-Acquah J.B. (2022). Improved approximated median filter algorithm for real-time computer vision applications. J. King Saud Univ.Comput. Inf. Sci..

[31] Otsu N. (1979). A Threshold Selection Method from Gray-Level Histograms. IEEE Trans. Syst. Man Cybern..

[32] Morton C.C. (2002). Genetics, genomics and gene discovery in the auditory system. Hum. Mol. Genet..

[33] Jacobs H.T., Hutchin T.P., Kappi T., Gillies G., Minkkinen K., Walker J., Thompson K., Rovio A.T., Carella M., Melchionda S. (2005). Mitochondrial DNA mutations in patients with postlingual, nonsyndromic hearing impairment. Eur. J. Hum. Genet. EJHG.

